# Structure of ThiM from Vitamin B1 biosynthetic pathway of *Staphylococcus aureus* – Insights into a novel pro-drug approach addressing MRSA infections

**DOI:** 10.1038/srep22871

**Published:** 2016-03-10

**Authors:** Julia Drebes, Madeleine Künz, Björn Windshügel, Alexey G. Kikhney, Ingrid B. Müller, Raphael J. Eberle, Dominik Oberthür, Huaixing Cang, Dmitri I. Svergun, Markus Perbandt, Christian Betzel, Carsten Wrenger

**Affiliations:** 1 University Hamburg, c/o DESY, Laboratory for Structural Biology of Infection and Inflammation, Hamburg, Germany; 2 Department of Biochemistry, Bernhard Nocht Institute for Tropical Medicine, Hamburg, Germany; 3 Fraunhofer Institute for Molecular Biology and Applied Ecology IME, Hamburg, Germany; 4 EMBL Hamburg, c/o DESY, Hamburg, Germany; 5 School of Life Sciences North Western Polytechnical University, Xi’an,Shaanxi, China; 6 Unit for Drug Discovery, Department of Parasitology, Institute of Biomedical Sciences, University of São Paulo, São Paulo, Brazil; 7 The Hamburg Centre for Ultrafast Imaging, Luruper Chaussee 149, D-22761 Hamburg, Germany

## Abstract

Infections caused by the methicillin-resistant *Staphylococcus aureus* (MRSA) are today known to be a substantial threat for global health. Emerging multi-drug resistant bacteria have created a substantial need to identify and discover new drug targets and to develop novel strategies to treat bacterial infections. A promising and so far untapped antibiotic target is the biosynthesis of vitamin B1 (thiamin). Thiamin in its activated form, thiamin pyrophosphate, is an essential co-factor for all organisms. Therefore, thiamin analogous compounds, when introduced into the vitamin B1 biosynthetic pathway and further converted into non-functional co-factors by the bacterium can function as pro-drugs which thus block various co-factor dependent pathways. We characterized one of the key enzymes within the *S. aureus* vitamin B1 biosynthetic pathway, 5-(hydroxyethyl)-4-methylthiazole kinase (*Sa*ThiM; EC 2.7.1.50), a potential target for pro-drug compounds and analyzed the native structure of *Sa*ThiM and complexes with the natural substrate 5-(hydroxyethyl)-4-methylthiazole (THZ) and two selected substrate analogues.

The ability of bacteria to adapt to antibiotic exposure led to the continuous development and spread of multi-drug resistant microbes causing severe infections and even death^1^. One of these pathogens is *Staphylococcus aureus (Sa)*, which is currently resistant against a broad range of the most useful antibiotics^2^,^3^. Despite the emergence of antibiotic resistance and relatively high mortality rates, the development of anti-infectives has seemingly been ignored for decades as a focus of drug discovery research investigations^4^,^5^,^6^. Most classical antibiotics inhibit cell wall linkage, interfere with the protein biosynthesis or decline nucleic acid synthesis. *Sa* developed strategies to obviate these impaired processes^7^. According to the WHO the 21^st^ century could become the post-antibiotic era and the World Health Assembly commissioned a global action plan in May 2014^8^. These and other worldwide governmental activities clearly highlight the urgent need for incentives to develop agents that can overcome acquired resistance to treat bacterial and in particular staphylococcal infections.

Ideally, new antibacterial drugs should specifically target metabolic processes that are exclusive to bacteria (like the vitamin B1 biosynthetic pathway, which is conserved among most bacteria, plants and lower eukaryotes^9^) but absent in humans. Humans have to rely on dietary uptake of thiamin and thus side effects of potential new antibiotics targeting this vitamin metabolism are in principle avoided^10^. Thiamin pyrophosphate (TPP), the active form of thiamin, is essential for the metabolism of carbohydrates and the amino acid metabolism^11^. In *Sa* six enzymes, ThiM, ThiD, ThiE, a nonspecific GTPase, TenA and TPK^12^, are involved in the pathway (see Supplementary Fig. S1) forming TPP. Only the 3D structure of TenA (PDB code: 4FN6) is known so far^13^, consequently more detailed structural information about the *Sa* thiamin biosynthesizing enzymes is required to support structure based drug discovery investigations.

Limiting the availability of this essential co-factor will thus effectively inactivate a diverse spectrum of bacterial targets and will cause a self-multiplying downstream effect. Hence targeting this metabolism is similar in concept to developing a suicide drug^14^. This approach uses a special mechanism closely related to the pro-drug concept but with an unique extension: it uncouples the drug’s sites of infiltration and action. As a result thiamin dependent pathways will be affected by the metabolized products and selective pressure will be moved from a single drug target towards several pathways depending on the activated form of thiamin.

Here we report a detailed structure-function analysis of the kinase ThiM, which phosphorylates THZ in one of the first steps in vitamin B1 biosynthetic pathway. In an attempt to achieve a maximum in accuracy and reliability of the structure, micro crystals grown under microgravity conditions onboard Chinese space mission Shenzhou-8^15^ were used to prepare seed stock solutions to grow crystals suitable for X-ray diffraction. In addition to the native structure, complexes with THZ and two selected lead compounds were analyzed.

The goal of these investigations was to identify potential pro-drug THZ analogues that do not inhibit ThiM, but will be accepted as substrates, yielding non-functional TPP analogues. In consequence these products cannot act as co-factors and the pathogen’s metabolic homeostasis will be disrupted, leading to energy imbalance, growth stoppage and, ultimately, death. As the sulfur atom in the thiazolium ring of thiamin is indispensable for the stabilization of a reactive carbanion at C2 position, forming an ylid-group, two THZ analogues with pyrazole and imidazole structure and lacking sulfur in the heterocycle were selected for comparative complex structure analyses and, in support of these studies, *in vitro* analyses of biochemical function were performed for them as well. It had already been shown that thiamin derivatives having an oxazolium or imidazolium ring are less reactive than thiazolium analogues, since in absence of 3d orbitals the transition state cannot be stabilized^16^,^17^,^18^. These comparative structural analyses of active site regions in combination with the data obtained from enzymatic assays yielded essential information to further design and optimize potential pro-drug THZ analogues.

## Results

### *Sa*ThiM structure

**Figure 1:**
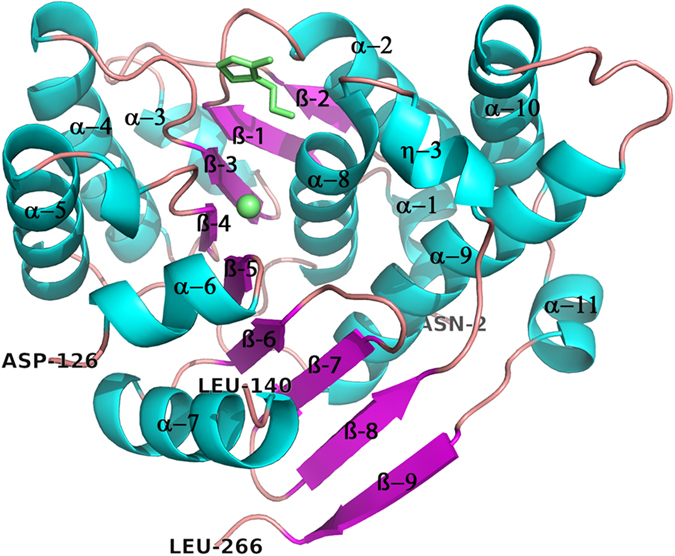
Overall structure of a ThiM monomer. Cartoon representation of the SaThiM monomer. Helices are coloured in cyan and ß-sheets in magenta. The active site region with a bound THZ and a magnesium ion are highlighted and colored in green.

**Figure 2:**
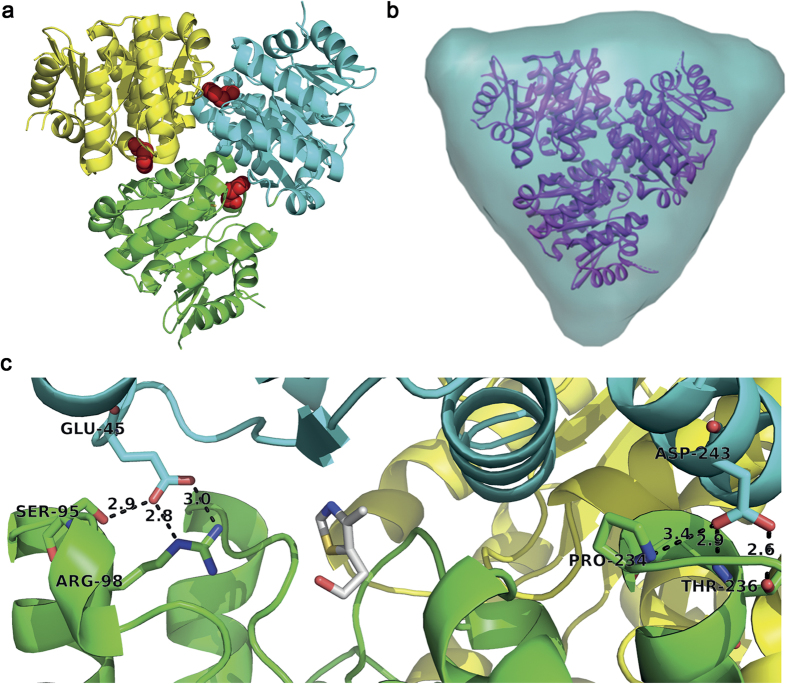
Structure of the ThiM trimer and its interface. (**a**) Cartoon representation of the *Sa*ThiM trimer, each monomer colored differently. The three active sites are located within the interface regions of the monomers, bound THZ molecules are shown as red spheres. (**b**) Front view of the *Sa*ThiM trimer *ab initio* shape in turquoise obtained from SAXS measurements and superimposed on the crystal structure, shown in a purple ribbon representation. (**c**) Detailed view to the interface and active site region with functional residues shown in stick representation with carbon chain atoms in the respective chain color, nitrogen in blue and oxygen in red. THZ is also shown in stick representation with carbon atoms in grey, nitrogen in blue and oxygen in red.

The quaternary structure of *Sa*ThiM is a triangular homo-trimer with dimensions of approximately 72 Å on a side and 47 Å thick with three independent active sites, each located within interface regions between two monomers (see Figs 1 and 2). The structure of native *Sa*ThiM was solved and refined to 2.1 Å resolution with R and R_free_ values of 20.34% and 23.04% respectively. Data collection and refinement parameters are summarized in Table 1 and the coordinates and data are available in PDB 5CM5. The homologue structure of *Bacillus subtilis* ThiK (PDB code: 1C3Q^19^), sharing 38% sequence identity, was used as a search model for molecular replacement phasing.

The secondary structure of *Sa*ThiM consists of approximately 50% α-helical and approximately 18% β-sheet structural elements (see Fig. 1). The core of each monomer contains nine β-sheets that are flanked by five α-helices and six α-helices. The first six β-sheets are parallel to each other with a corresponding strand order 2-1-3-4-5-6. The following β-sheet strand 7 is antiparallel to strand 6 and following β-sheet strand 8, which is antiparallelly flanked by β-sheet strand 9. This fold of the phosphotransferase ThiM is homologous to that common to the group of ribokinase-like folded kinases^20^. For all six molecules in the asymmetric unit no electron density was observed for the last two residues of the C-terminus and for the loop region between residues 126 and 140. Therefore these residues were not modeled. Analysing the interface regions of protomers A, B and C revealed that 10 intermolecular H-bonds support the complex formation. Analysis was done by manual inspection using the program PDBsum^21^. All intermolecular interactions are summarized in Supplementary Tables S1 and S2. The total surface of one monomer was calculated to be approximately 11,500 Å^2^ with almost 1,000 Å^2^ buried upon assembly of two monomers. The ThiM trimer has approximately 27,000 Å^2^ solvent accessible surface area, and approximately 5,700 Å^2^ of the monomeric surface accessible area is buried upon quarternary structure formation (see Fig. 2).

The trimeric assembly observed in the crystal structure was validated in solution with small angle X-ray scattering (SAXS). The experimental radius of gyration R_g_ of 30 ± 2 Å, the maximum dimension D_max_ of 90 ± 10 Å and the shape of the p(r) function (Supplementary Fig. S2), point to a globular structure. The protein molecular mass estimated from the Porod volume and from the *ab initio* model built with the program DAMMIF^22^ are 91 ± 8 kDa and 77 ± 8 kDa respectively, which is compatible with trimeric *Sa*ThiM in solution. The DAMMIF model with enforced P3 symmetry is well superimposable with the crystal structure and resembles the trimeric structure in solution, as shown in Fig. 2b. The scattering computed from the trimeric model where the missing residues were symmetrically reconstructed with the program CORAL^23^ fits the experimental SAXS data well with χ^2^ = 0.6 (see Supplementary Fig. S2). All SAXS-derived parameters are summarized in Table 2, the scattering data and the models are deposited in SASBDB^22^, code: SASDAX8.

**Figure 3:**
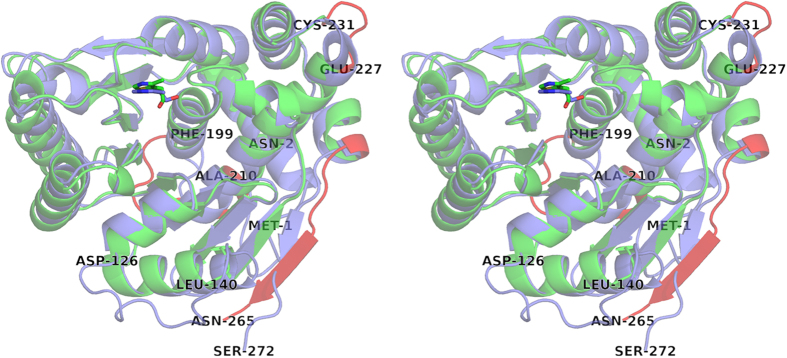
Superimposed structures of SaThim and BsThiK. Stereo view of the superimposed homologues structures of *Sa*ThiM (colored in green) and ThiK from *Bacillus subtilis* (colored in blue) in a cartoon representation. The regions with higher differences, the loop region from residues 199–210 connecting helix 8 and 9 and close to N- and C-termini, are colored in red. The loop regions 126–140 are not modeled into *Sa*ThiM structure.

A sequence alignment of ThiM with homologous structures by means of a NCBI/Blast PDB search option showed only a moderate degree of structural conservation among ThiM-type enzymes in bacteria (see Supplementary Fig. S3). *Sa*ThiM shares 32% amino acid sequence identity with *E. faecalis* ThiM (PDB code: 3DZV), 38% with *B. subtilis* ThiK (PDB code: 1C3Q) and 39% identity with *P. horikoshii* ThiM (PDB code: 3HPD). The corresponding Cα r.m.s.d. values are 1.6, 1.3, and 1.2 Å and maximum displacements were calculated to be 4.6, 4.5 and 4.2 Å, using the protein structure comparison service Fold at the European Bioinformatics Institute^24^. The overall fold of the structures is conserved, whereas higher differences were found in the loop regions from residues 126–140 (connecting helix 6 and 7), 199–210 (connecting helix 8 and 9), 227–231 (connecting helix 9 and 10) and the regions close to N- and C-termini (see Figs 1 and 3). In *Sa*, part of the interface stabilization is conserved and mediated through inter-protomer H-bonds: E45 forming three H-bonds to R98, and S95 and D243 forming three H-bonds to P234 and T236 in the neighboring monomer (see Fig. 2c). A comparison of homologous hydroxyethylthiazole kinases from *B. subtilis* and *P. horikoshii* shows that this pattern of stabilization in the interface regions of the trimeric assembly, with two defined positions with amino acids forming two H-bonds to two neighboring amino acids, is highly conserved.

### Active site and enzyme kinetics

**Figure 4:**
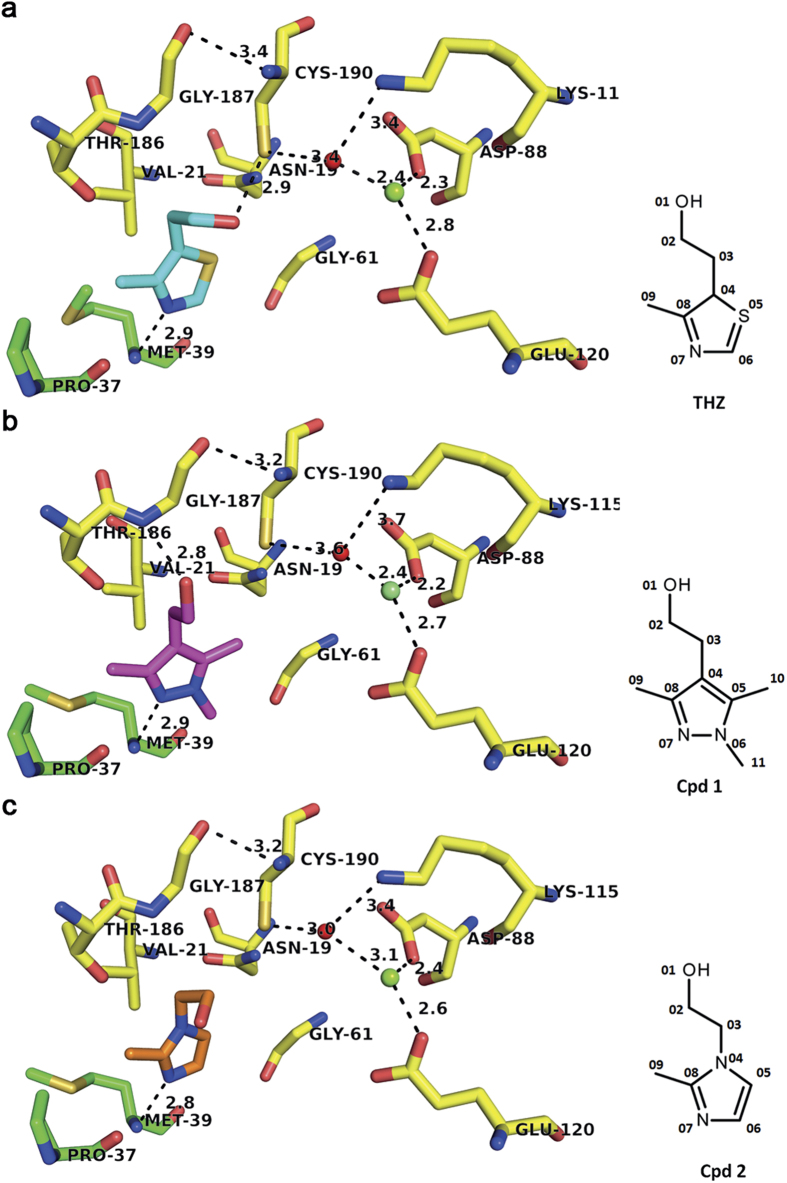
*Sa*Thim active site with bound substrates. *Sa*ThiM active site with bound THZ (**a**), cpd 1 (**b**) and cpd 2 (**c**). The active site residues are shown in stick representation with carbon chain atoms in the respective chain color, nitrogen in blue, oxygen in red and sulphur in yellow. For THZ carbon atoms are colored in cyan, for cpd 1 in magenta and for cpd 2 in orange. Waters are displayed as red and Mg^2+^ ions as green spheres. H-bonds are shown as dashed lines. Consensus nomenclature of the substrate THZ and selected lead compounds cpd 1 and cpd 2 are shown, respectively.

Based on the crystal structure in complex with the natural substrate THZ, refined to 1.90 Å resolution with R and R_free_ values of 18.61% and 20.95% respectively (Table 1, PDB: 5COJ), the three identical active sites of the trimer were characterized and the mode of action for phosphorylation could be proposed. In the crystal structure, THZ identically occupies all six active sites of the two trimers in the asymmetric unit. A view of the experimental electron density of ThiM in complex with THZ can be found as Supplementary Fig. S4. THZ binding is mediated by interactions with amino acids N19, V21, G61, V90, T186, G187 and C190 of one monomer and P37, A38 and M39 of the corresponding neighboring monomer, all located in the interface region. Substrate binding is mainly stabilized by H-bonds formed by the nitrogen of the heterocycle and via the flexible hydroxyl group of THZ (see Fig. 4a). The orientation of the hydroxyl group, as extracted from the THZ complex formation in all six active sites in the asymetric unit, can adopt slightly different orientations (see Supplementary Table S3). In summary THZ forms six direct H-bonds to residues M39, P37, G61, T186 and C190. For the ThiM-THZ complex four Mg^2+^ ions, essential for the phosphor transfer reaction, were located within four active sites of the two trimers in the asymmetric unit. The Mg^2+^ ions are coordinated by residues D88, K115 and E120 and one solvent water molecule as shown in Fig. 4a. No Mg^2+^ ion was found in the native structure. Utilizing also the structural data obtained from homologous ThiM-complex structures, such as the mutated ThiK homologue, from *Bacillus subtilis*^19^ (PDB code: 1ESQ) with bound ATP and THZ, as well data known about conserved phosphoryl transfer within ribokinases-like kinases^25^, the following mode of action can be proposed, which is additionally illustrated in Supplementary Fig. S5. Enhanced nucleophilicity of the THZ alcohol is expected due to a proton-relay system consisting of a water molecule, which is coordinated by a magnesium ion and C190. Additionally two oxygen atoms of the ATP-γ-phosphate are coordinated by the magnesium ion and N117 resulting in a enhanced electrophilicity of the γ-phosphate and a pentavalent intermediate between the γ-phosphate and THZ. Further, the transition state is stabilized by hydrogen bonds of T160 OG to the α-phosphate and of K115 NZ to the β-phosphate.

In order to identify substrate analogues for *Sa*ThiM, a structure-based virtual screening approach was conducted. At first, a focused compound library was generated by searching the ZINC database^26^ for THZ structure analogues by means of a SMARTS query containing several restrictions: i) a 5-membered heterocyclic ring including a nitrogen atom to enable hydrogen bonding with N39 and excluding a sulfur atom in order to inhibit the carbanion formation for thiamin-dependent enzymes^27^, and ii) a hydroxy-ethyl moiety in position 4 of the 5-membered ring to allow phosphorylation by the enzyme. Applying these constraints resulted in 78 compounds that were docked into the *Sa*ThiM active site using AutoDock 4.2.3^28^ with default settings. Based on docking scores and visual analysis of compound-binding modes, 9 compounds were identified as putative *Sa*ThiM substrates. Out of this *in silico* selected set of molecules, two were chosen as the most promising analogues for further investigations: 2-(1,3,5-Trimethyl-1H-pyrazol-4-yl) ethanol, hereinafter referred to as compound 1 (cpd 1) and 2-(2-Methyl-1H-imidazol-1-yl) ethanol referred to as compound 2 (cpd 2). The activity of ThiM was analyzed *in vitro*, as described in the methods section, showing that the specific activities of cpd 1 and cpd 2 are approximately 1.5 times higher compared to THZ, however show only 1/10 of the efficiency (k_cat_/K_M_) in comparison to the natural substrate THZ (see Table 3 and Supplementary Fig. S6). This is also reflected by the higher K_M_-value of the compounds.

In summary the kinetic data confirmed that the selected compounds were accepted as substrates, even if *in vitro* turnover was slightly lower, and could thus be used for further investigations and to optimize effective pro-drug compounds targeting the vitamin B1 biosynthetic pathway. Based on these findings complexes of *Sa*ThiM with cpd 1 and cpd 2 were prepared and crystallized.

The structures of these complexes could be refined to 1.87 Å and 1.62 Å resolution with R and R_free_ values of 16.86% and 19.90% for the cpd 1 complex and R and R_free_ values of 18.24% and 20.28% for the cpd 2 complex. Data collection and refinement parameters are summarized in Table 1 and the coordinates for the structures, as well as the experimental diffraction amplitudes have been deposited at the Protein Databank (http://www.rcsb.org) with entry codes 5CGA and 5CGE. The chemical structure as well as the compounds bound in the active site are shown in Fig. 4b,c, respectively. The overall coordination of the THZ analogue compounds in the active site regions of the ThiM trimer is remarkably comparable to that of the of natural substrate THZ (see Fig. 4a). Both compounds form a H-bond to the amide nitrogen of amino acid M39 and show a similar orientation of their hydroxyethyl groups. The thiazole moiety was replaced by the non-sulfur containing heterocycles pyrazole and imidazole for cpd 1 and cpd 2, respectively. Moreover, cpd 1 contains two additional methyl groups at position 5 and 6 of the heterocycle (see Fig. 4b,c, respectively). These structural differences to the natural substrate result in a 1.5 higher specific activity for both cpd 1 and cpd 2, even if the location of the compounds in the active site is highly comparable to that of the natural substrate, as shown in Table 3, confirming the appropriate pre-positioning for the phosphoryl transfer reaction.

## Discussion

The shortfall in the development of new antibiotics and the misguided overuse of the currently available antibiotics has caused an unfortunate spread of multi-resistant bacteria in recent years. Because of this, rational structure-based drug discovery is urgently required^6^ to cover the loss by supporting accelerated design of highly selective antibiotics with an elaborated mode of action. To our knowledge this study represents the first approach in targeting the *Sa* thiazole kinase ThiM of the bacterial vitamin B1 pathway, applying structure based pro-drug discovery. In order to improve crystal quality of *Sa*ThiM, we performed crystallization trials under microgravity conditions in combination with micro seeding techniques. This procedure has previously been applied successfully to optimize crystal quality^29^. We observed an increase in crystal quality compared to native triclinic *Sa*ThiM crystals obtained in previous experiments^30^. Soaking of the respective crystals with ThiM substrate and selected pro-drug compounds resulted in loss of crystal symmetry. Similar observations were made for crystals of the ThiM homologue ThiK from *B. subtilis*, where soaking with substrates led to crystalline complexes with monoclinic symmetry, in comparison to native rhombohedral crystals^19^. This effect could be attributed to a slightly changed fold in the loop region 126–140, which changed the interactions and crystal packing of the trimeric subunits in the asymmetric unit. *Sa*ThiM revealed a conserved ribokinase-like fold and a trimeric assembly, which was confirmed by complementary SAXS experiments. The three active sites, each located within the interface regions of two monomers respectively, are homologous to other known phosphoryl transfer ribokinase-like kinases^25^. Based on the overall structural information of ThiM complexes obtained, we propose a catalytic pathway of *Sa*ThiM. Further, we identified the role of C190 and the coordinated magnesium ion in enhancing the nucleophilicity of the THZ alcohol and thereby facilitating its phosphorylation via a proton relay mechanism. Structural data of THZ bound in the active sites of *Sa*ThiM allowed *in silico* screening and identification of two initial lead compounds. In terms of the pro-drug development concept, these compounds were used to prepare enzyme-compound complexes. The results of the biochemical assays, complemented by the structural data, support the suggested pathway of phosphorylation. Comparing the specific activities of ThiM in presence of the two compunds revealed a similar turnover as that of the natural substrate THZ, which clearly validates the approach to combine complementary methods for pro-drug discovery in order to identify and improve lead compounds.

The two novel compounds applied for co-crystallization in this study lack the thiazolium sulfur, present in the natural thiazolium moiety of thiamin, which is replaced by a carbon atom, supporting slightly higher flexibility in the active site in consequence of the reduced van der Waals radius. Overall, the orientation of both compounds in the active sites of ThiM is very similar to that observed for the natural substrate THZ.

For both compounds the N3 is retained and a second nitrogen atom at position 2 and 5 is included in the heterocycle of cpd 1 and cpd 2 respectively. Moreover, cpd 1 has two additional methyl groups bound to atoms 1 and 2, which can provide more hydrophobic interactions in binding co-factor dependent enzymes after complexation to the HMP moiety by ThiE thereby enabling the cpd 1-HMP moiety to act as a competitive inhibitor. Cpd 2 has an additional nitrogen atom in the heterocycle and could, like cpd 1, undergo a TPP imino tautomerization after fusion with HMP. As a result this substance could be channeled via thiaminase (TenA) into the bacterial thiamin pool, where it could act as a thiamin antagonist equally potent as pyrithiamine^31^. The data presented here summarize the first steps towards the development of thiamin analogue compounds, only produced *in vivo* in bacteria cells, which can inhibit preferably multiple co-factor dependent enzymes and in addition might also block the thiamin riboswitch in the bacteria^32^. This approach will open a route for the development of new antimicrobial substances to treat MRSA infections.

## Methods

### Purification and Crystallization

The expression, purification and initial crystallization of native *Sa*ThiM has been described previously^30^. To optimize the crystal quality and try for a better crystal packing, crystallization experiments under microgravity conditions were performed in the DLR SIMBOX experiment^15^,^33^ onboard the Chinese space mission Shenzhou-8^15^. The SIMBOX counter diffusion crystallization experiments were coordinated by the Institute of Biophysics of the Chinese Academy of Sciences, Beijing. For the space experiment, protein solutions were dialyzed overnight against 100 mM Tris, 150 mM NaCl, pH 8.0. ThiM was concentrated to approximately 20 mg/mL, an equal volume of 0.5 M magnesium formate was added at 4 °C, incubated for at least 6 h and centrifuged at 20,000 × g for 30 min. Prior to the microgravity experiments, crystallization conditions were extensively optimized under laboratory conditions, including transport simulations. The final protein solutions were analyzed by dynamic light scattering and showed a stable monodisperse peak for several days. The capillary length of the SIMBOX crystallization hardware was 10 mm with an inner diameter of 1 mm. The capillary was filled with a ThiM solution using a concentration of 12 mg/mL and 2% (w/v) low melting point agarose (Serva) was used to seal the capillaries. Subsequently, the capillaries were inserted carefully into a plastic casing, containing four separated sections filled with foam material and sealed with a metal lid. The foam was saturated with approximately 300 μL of precipitant solution (20% PEG 3,350 (w/v), 7% isopropanol (v/v)). For the microgravity experiment 4 capillaries were used and positioned in the DLR SIMBOX. The microgravity experiment was performed at a stable temperature of 20 °C for 17 days (30.10.2011–17.11.2011) onboard the Shenzhou-8 space mission. After return of the experiment the capillaries were inspected directly after arrival in Beijing. The capillaries were opened at one end and the agarose was removed carefully. Obtained micro-crystals were used to prepare native ThiM and complex crystals applying hanging drop vapor diffusion and a streak seeding procedure^34^. Seed stocks were prepared following the manufacturer’s instructions (Jena BioScience Beads-for-seeds). 24-well CPL-130 plates (Jena Bioscience) for hanging drop vapor diffusion experiments were filled with 500 μl of precipitant solution and ThiM was used at a concentration of 10 mg/mL. In 48-well MRC sitting drop plates (Molecular Dimensions) for sitting drop vapor diffusion, two rows of the precipitants with different PEG 3,350 concentrations were prepared by filling 50 μl of precipitant (18, 20 and 22% PEG 3,350 (w/v), 0.2 M magnesium formate and 5% isopropanol (v/v)) into the reservoirs. Purified ThiM was dialyzed overnight against 50 mM Tris (pH 8.0), 150 mM NaCl and directly used at 10 mg/mL concentration after centrifugation (100,000 × g, 4 °C for 50 min). To obtain a ThiM-THZ complex native crystals were supplemented with THZ at a concentration of 0.5 mM overnight. To obtain ThiM-cpd 1 and ThiM-cpd 2 complexes, crystals were soaked with a 25 mM and 20 mM compound 1 und 2 solutions respectively overnight. Prior to data collection crystals were soaked in precipitant solution containing 10% v/v glycerol as cryoprotectant and THZ, cpd 1 or cpd 2 respectively.

### Structure analysis

Diffraction data of native ThiM were collected as described before^30^. A complex crystal of ThiM-THZ was used to collected diffraction data using a MARCCD165 detector at the DORIS consortium beamline X13 (HASYLAB/DESY) using a wavelength of 0.81 Å at 100 K. Diffraction data of ThiM-cpd 1 and ThiM-cpd 2 crystals were collected at the EMBL beamline P14 (PETRAIII, DESY, Hamburg) at 0.98 Å wavelength and 100 K with a PILATUS 6M detector.

Data processing and scaling of native ThiM and ThiM-THZ were carried out using DENZO and SCALEPACK^35^, ThiM-cpd 1 and ThiM-cpd 2 data sets were processed applying the program XDS^36^. The uncomplexed crystals belonged to the space group P2_1_ (Unit cell parameters: a = 62.6 Å, b = 103.5 Å, c = 126.2 Å and β = 99.5°) with six ThiM molecules, forming 2 trimers, in the asymmetric unit. The packing parameter V_M_ was calculated to be 2.3 Å^3^/Da^37^, which corresponded to a solvent content of 47%. Native ThiM crystals, which were grown applying microgravity grown seed stock crystals, were in the monoclinic space group P2_1_. Soaked THZ, cpd 1 and cpd 2 complex crystals are assigned to the triclinic space group P1, due to slightly changed crystal contacts of the six ThiM molecules in the unit cell. Unit cell parameters and data collection and scaling statistics are summarized in Table 1. The coordinates of *Bacillus subtilis* ThiK (PDB code: 1C3Q) were used as a starting model for molecular replacement applying the program PHASER^38^ within the CCP4 suite^39^. The refined native ThiM structure was subsequently used as a starting model for the complex structures, ThiM-THZ, ThiM-cpd 1 and ThiM-cpd 2 applying MOLREP^40^. The models were refined through iterative cycles of restrained refinement by REFMAC5^41^ and phenix.refine^42^ and manual rebuilding applying the program COOT^43^.

Resolution limits of the diffraction data were set applying the criteria I/σ > 2, monitoring R_merge_ with a limit of approximately 50%. These criteria resulted in resolution limits of 2.1 Å of the native diffraction data set, 1.9 Å for the THZ complex, 1.85 Å for the cpd 1 and 1.6 Å for the cpd 2 complex. After first manual model building and refinement solvent molecules were included applying Fo-Fc difference maps. For the complex crystals the THZ, cpd 1 and cpd 2 moieties were introduced and refined. Optimized geometry restraints for bond distance and angles of THZ as well as for cpd 1 and cpd 2 were generated with Phenix eLBOW^42^ and applied for refinement. Magnesium ions, if present, were positioned applying Fo-Fc density maps contoured at 2.5 σ in a tetragonal coordination with ligand residues L115, E120 and D88.

For native ThiM 98% of residues are in the favoured, 2% are in the additional allowed regions of the Ramachandran plot and no residue was found in the disallowed regions. For ThiM-THZ, ThiM-cpd 1 and ThiM-cpd 2 99% are in the favoured regions and 1% is in the additional allowed regions of the Ramachandran plot and no residue was found in the disallowed regions. Analysis was done with MolProbity^44^.

### Small angle X-ray scattering

SAXS data of native ThiM were collected at the EMBL beamline X33 (DORIS III, DESY, Hamburg)45. Using a 1M-W PILATUS detector at a sample-to-detector distance of 2.7 m and a wavelength of λ = 1.5 Å, the range of momentum transfer 0.01 < s < 0.6 Å-1 was covered (s = 4π sinθ/λ, where 2θ is the scattering angle). Four solute concentrations ranging between 1.3 and 9.9 mg/ml were measured at 12 °C. To monitor for the radiation damage, eight successive 15-second exposures of protein solutions were compared and no statistically significant changes were detected. The data were normalized to the intensity of the transmitted beam and radially averaged; the scattering of the buffer was subtracted and the difference curves were scaled for protein concentration. The low angle data measured at lower protein concentrations were merged with the higher concentration data to yield the final composite scattering curves. The radius of gyration Rg, the Porod volume of the hydrated particle along with the distance distribution function p(r) and the maximum intra-molecular distance Dmax were derived using the automated SAXS data analysis pipeline SASFLOW46. The molecular mass (MM) was obtained from the Porod volume: for globular proteins Porod volumes in Å^3^ are about 1.6 times the MMs in Da. *Ab initio* shape model was reconstructed using the bead modelling program DAMMIF^45^. This program represents the particle shape by an assembly of densely packed beads and employs simulated annealing to construct a compact interconnected model fitting the experimental data. A molecular mass estimate is derived from the volume of the model. Ten DAMMIF runs were performed to check the stability of solution, and the results were well superimposable with each other. 15 C-terminal residues missing from the high-resolution X-ray structure were reconstructed with the rigid body modelling program CORAL^23^, the fit to the experimental data was refined with CRYSOL^46^. All SAXS-derived parameters are summarized in Table 2, the scattering data and the models are deposited in SASBDB^22^, code: SASDAX8.

### Activity analysis

*Sa*ThiM activity assays were performed in 100 μl buffer solution containing 50 mM potassium phosphate (pH 7.5), 10 mM MgCl_2_, 4 mM ATP (supplied by Sigma), 50 nCi ATP-[P^33^] and 0–4 mM THZ. 315 ng of purified protein was added and incubated for 15 min at 37 °C. Reactions were quenched at 95 °C for 2 minutes and reaction products were analyzed as described before^12^. *De novo* synthesized THZ-[P^33^] was quantified using a LS5000 CE scintillation detector (BeckmanCoulter). The kinetic parameters were calculated using the GraphPad Prism 5 software (GraphPad Software) as described previously^47^ and summarized including the standard deviation in Table 3 and as Supplementary Fig. S6.

## Additional Information

**Accession codes:** PDB: 5CM5 (native), 5COJ (THZ complex), 5CGA (cpd 1 complex) and 5CGE (cpd 2 complex); SASBDB_23_ code: SASDAX8. 

**How to cite this article**: Drebes, J. *et al.* Structure of ThiM from Vitamin B1 biosynthetic pathway of *Staphylococcus aureus* – Insights into a novel pro-drug approach addressing MRSA-infections. *Sci. Rep.* **6**, 22871; doi: 10.1038/srep22871 (2016).

## Supplementary Material

Supplementary Information

## Figures and Tables

**Table 1:** Data collection and refinement statistics for native *Sa*ThiM, *Sa*ThiM in complex with THZ and in complex with two substrate analogues, cpd 1 and cpd 2.

	ThiM native	ThiM-THZ	ThiM-cpd 1	ThiM-cpd 2
Data collection				
Space group	P2_1_	P1	P1	P1
Cell dimensions				
*a*, *b*, *c* (Å)	62.6, 103.5, 126.2	62.6, 62.7, 108.5	62.0, 62.4, 108.3	62.4, 62.5, 109.2
α, β, γ (°)	90.0, 99.5, 90.0	92.2, 91.4, 101.3	92.6, 91.4, 101.3	92.6, 92.1, 101.5
Resolution (Å)	20.0–2.09	20.0–1.90	30.0–1.87	30.0–1.62
*R*_merge_	7.8 (49.2)	2.7 (21.1)	6.9 (54.9)	6.9 (51.3)
*I*/σ	17.3 (4.2)	21.8 (4)	12.7 (2.5)	10.7 (2.5)
Completeness (%)	99.9 (100)	94.7 (92.9)	95.8 (94.7)	93.4 (90.5)
Redundancy	7.8 (7.7)	2 (1.9)	3.6 (3.5)	3.6 (3.6)
Refinement				
Resolution (Å)	20.0–2.09	20.0–1.90	30.0–1.87	30.0–1.62
No. reflections	88976	117078	119598	181567
*R*_work_/*R*_free_	20.34/23.04	18.61/20.95	16.86/19.90	18.24/20.28
No. atoms				
Protein	11474	11149	11228	11453
Ligand	–	54	66	54
Ions	–	4 Mg^2+^	4 Mg^2+^	5 Mg^2+^
Water	552	371	486	511
*B*-factors				
Protein	39.1	41.1	37.5	30.5
Ligand	–	43.2	38.1	24.9
Ion	–	41.2	44.8	43.6
Water	33.4	26.0	33.8	26.1
R.m.s. deviations				
Bond lengths (Å)	0.016	0.017	0.019	0.017
Bond angles (°)	1.615	1.598	1.738	1.699

*Values in parentheses are for highest-resolution shell.

**Table 2:** SAXS data collection and derived parameters.

Data collection parameters	
Beamline	EMBL beamline X33 (DORIS III, DESY)^48^
Wavelength (Å)	1.5
s range (Å^−1^)	0.01–0.6
Exposure time (s)	120
Concentration range (mg/ml)	1.3–9.9
Temperature (°C)	12
Structural parameters	
R_g_ (Å) (from Guinier)	30 ± 2
R_g_ (Å) (from p(r))	29 ± 3
D_max_ (Å)	90 ± 10
Porod volume estimate (Å^3^)	145, 000 ± 15, 000
Molecular mass from Porod volume (kDa)	91 ± 9
Molecular mass from dummy atom modelling (kDa)	77 ± 8
Monomeric molecular mass calculated from sequence (kDa)	29.8
Software employed	
Primary data reduction	SASFLOW pipeline^49^
*Ab initio* analysis	DAMMIF^45^
Rigid body modelling	CORAL^23^

**Table 3:** Kinetic parameters of *Sa*ThiM phosphorylating THZ^12^, cpd 1 and cpd 2.

	Specific Activity [nmol/min/mg]	K_M_ [μM]	k_cat_ [min^−1^]
THZ^12^	4880 ± 488	44 ± 5	137 ± 13
cpd 1	7297 ± 267	834 ± 147	215 ± 8
cpd 2	7418 ± 91	831 ± 169	218 ± 3
