# Supplementary material for: Structure of ThiM from Vitamin B1 biosynthetic pathway of Staphylococcus aureus – Insights into a novel pro-drug approach addressing MRSA infections
Source: Sci Rep. 2016 Mar 10;6:22871. doi: 10.1038/srep22871 (PMC4785402; doi:10.1038/srep22871)
Supplement: Supplementary Information [file srep22871-s1.pdf]

Supplementary Information

Structure of ThiM from Vitamin B1 biosynthetic pathway of *Staphylococcus aureus* – Insights into a novel pro-drug approach addressing MRSA infections

Julia Drebes[a,b,#], Madeleine Künz[a,#], Björn Windshügel[c], Alexey G. Kikhney[d], Ingrid B. Müller[b], Raphael J. Eberle[a,‡], Dominik Oberthür[a,§], Huaixing Cang[e], Dmitri I. Svergun[d], Markus Perbandt[a,g], Christian Betzel[a,g,*] and Carsten Wrenger[f,*]

[a] University Hamburg, c/o DESY, Laboratory for Structural Biology of Infection and Inflammation, Hamburg, Germany

[b] Department of Biochemistry, Bernhard Nocht Institute for Tropical Medicine, Hamburg, Germany

[c] Fraunhofer Institute for Molecular Biology and Applied Ecology IME, Hamburg, Germany

[d] EMBL Hamburg, c/o DESY, Hamburg, Germany

[e] School of Life Sciences North Western Polytechnical University , Xi'an,Shaanxi, China

[f] Unit for Drug Discovery, Department of Parasitology, Institute of Biomedical Sciences, University of São Paulo, São Paulo, Brazil

[g] The Hamburg Centre for Ultrafast Imaging , Luruper Chaussee 149 , D-22761 Hamburg, Germany.

[#] These authors contributed equally to this work

[*] To whom correspondence should be addressed: Christian.Betzel@uni-hamburg.de or cwrenger@icb.usp.br

[‡] Present address: Multiuser Center for Biomolecular Innovation, Department of Physics,

Universidade Estadual Paulista (UNESP), São Jose do Rio Preto-SP 15054-000,

Brazil.

[§] Present address: Center for Free-Electron Laser Science, Deutsches Elektronen Synchrotron-DESY, Notkestrasse 85, 22607 Hamburg, Germany

Supplementary tables:

Table S1: Structural details of the interface regions.

Chains	Nr. of residues in the interface	Interface areas (Å^2)	Nr. of H- bonds	Nr. of hydrophobic interactions
A-B	19:19	1067:1037	10	117
B-C	18:18	953:926	10	116
C-A	18:18	941:906	10	107

Table S2: Hydrogen bonds in the interfaces.

	Atom Interface 1	***Atom Interface 2***
1	K23 NZ	D20 OD2
2	H247 NE2	G232 O
3	E45 OE1	S95 OG
4	E48 OE1	T96 OG1
5	E45 OE1	R98 NE
6	E45 OE2	R98 NH2
7	D243 OD1	P234 N
8	D243 OD1	G235 N
9	D243 OD1	T236 N
10	D243 OD2	T236 OG1

Table S3:
ThiM -THZ, -cpd 1 and -cpd 2 direct H-bond-interactions. THZ and cpd consensus nomenclature as declared in Figure 4.

		**THZ**		**cpd 1**		**cpd 2**	
ThiM amino acid and atom	THZ/cpd molecule consensus nomenclature	Mean distance [Å]	Standard deviation [Å]	Mean distance [Å]	Standard deviation [Å]	Mean distance [Å]	Standard deviation [Å]
G187 N	01	-	-	2.8	0.11	-	-
M39 N	07	2.9	0.14	2.9	0.10	2.8	0.05
C190 SG	01	2.9	0.85	-	-	-	-

Supplementary figures:

Fig. S1: Vitamin B1 pathway

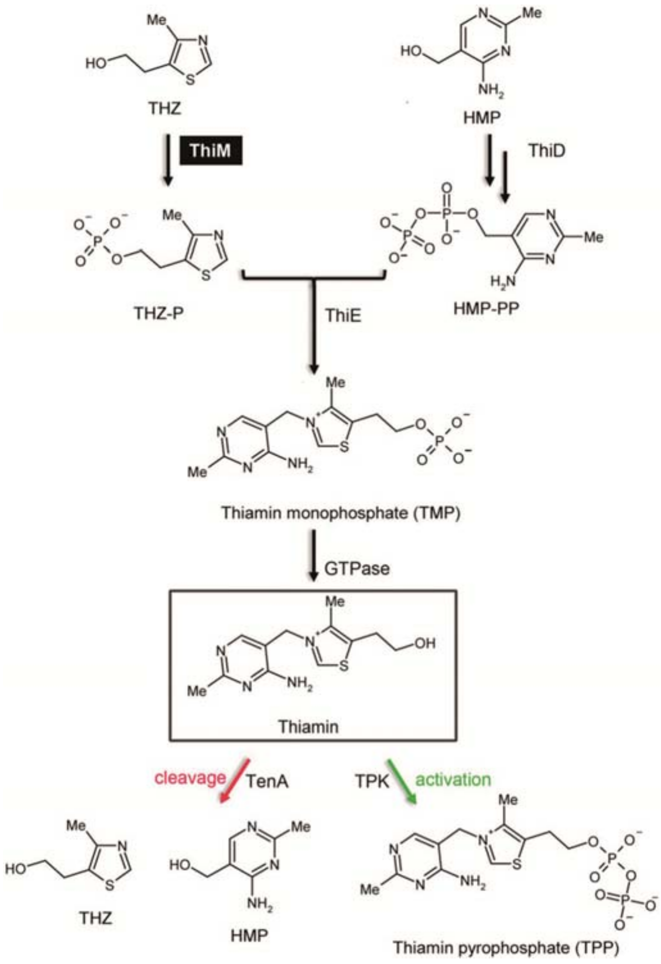

Scheme of the *Sa* vitamin B1 biosynthetic pathway according to Müller *et al.,* 2009[11]*.* ThiM phosphorylates THZ in an early stage of thiamin biosynthesis and enables coupling of the thiazole moiety to the pyrimidine moiety.

Fig. S2: Experimental SAXS data

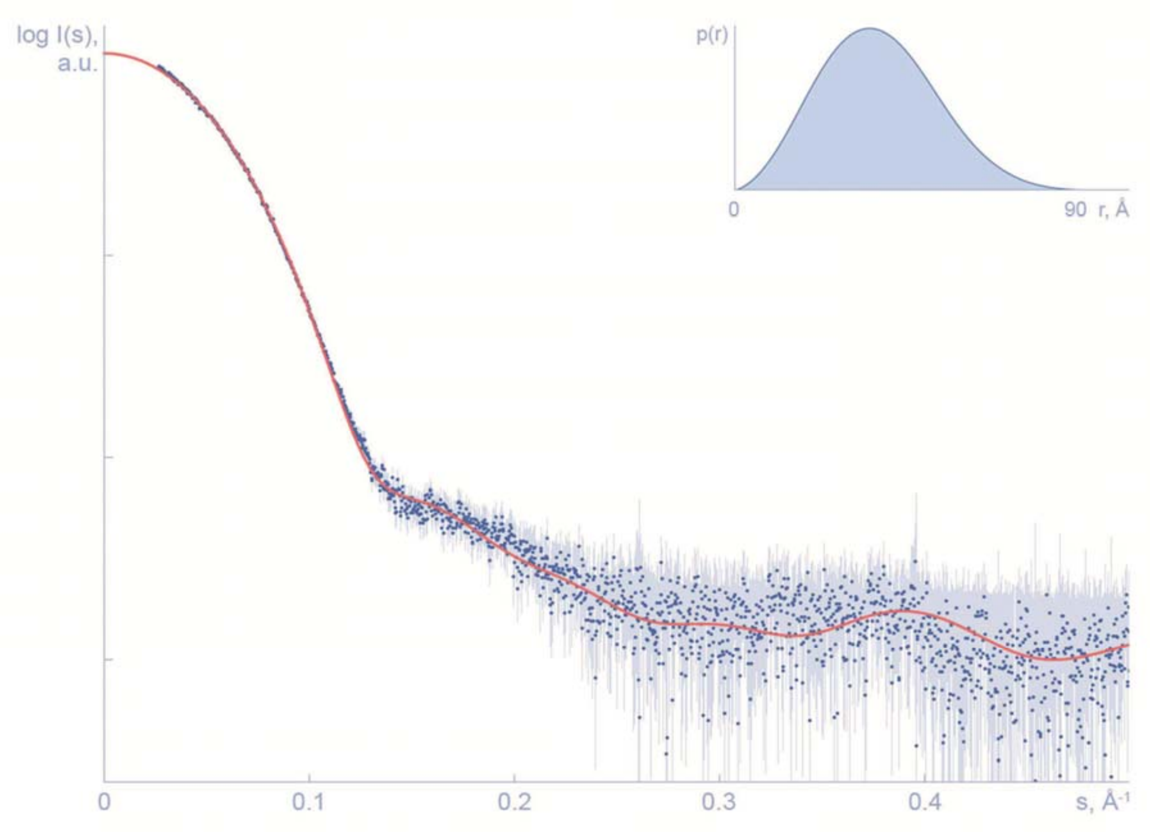

Processed SAXS data from *Sa*ThiM (blue dots with error bars) and the fit calculated from the trimeric model with reconstructed missing residues (red line). Inset: pair distance distribution function p(r).

Fig. S3: Sequence aligment with homologue structures

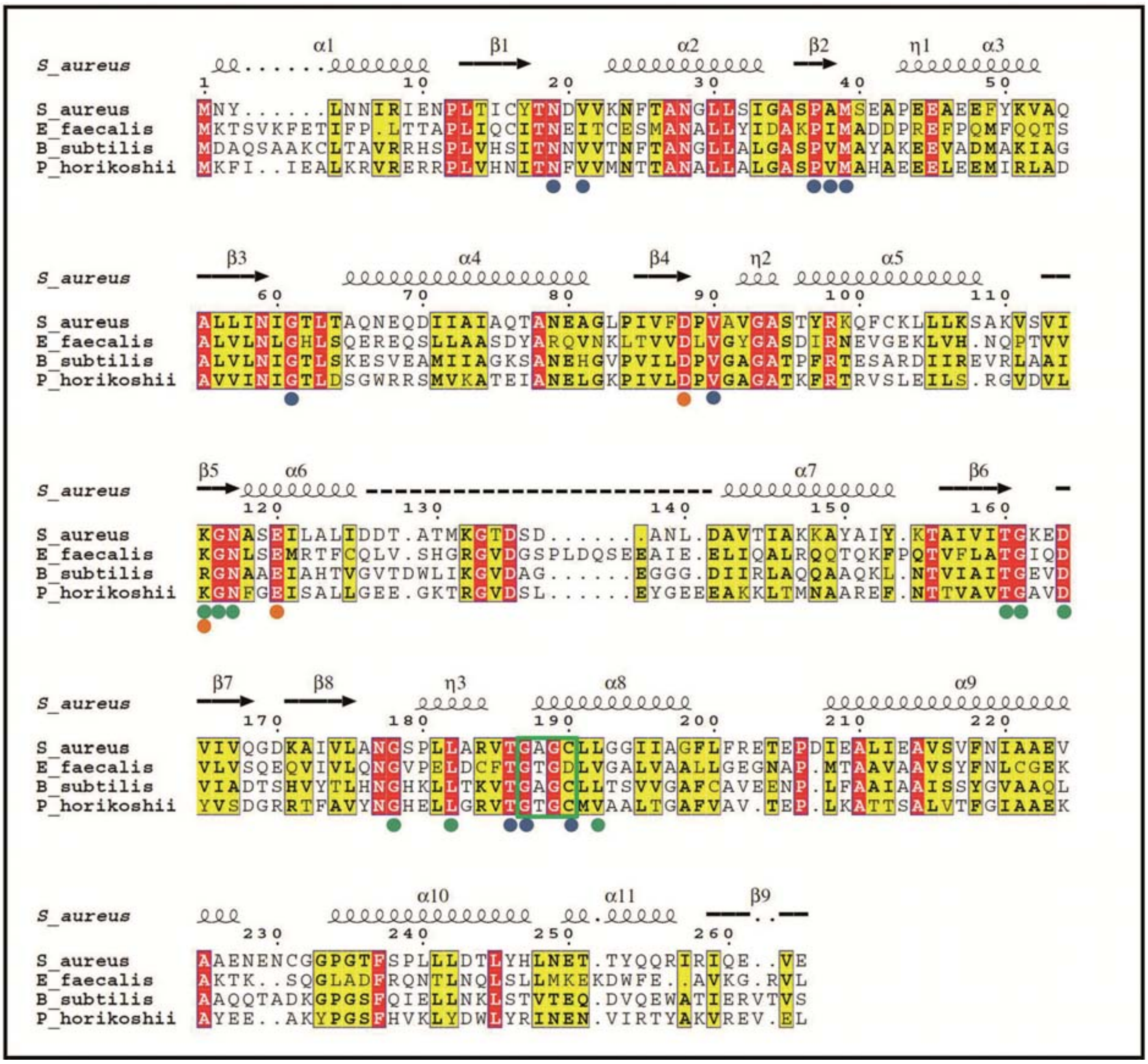

*Sa*ThiM sequence alignment towards the homologue structures of *E. faecalis, B. subtilis* and *P. horikoshii*, carried out using Clustal W[50] and ESPript 3.0[51]. Identical residues are boxed in red and similar residues in yellow. Secondary structure elements, α-helices, β-strands and 3_{10}-helical elements are indicated and labled with α, β and η and consecutive numbering. Dashed lines indicate a disordered region. The catalytic region is framed in green. Blue circles below the sequence indicate THZ and cpd 1 and cpd 2 binding residues. Green dots indicate the ATP-binding site as found for the homologous structure ThiK (PDB code: 1ESQ) of *B. subtilis* and orange dots indicate residues binding Mg^{2+}.

Fig. S4: Representative electron density map

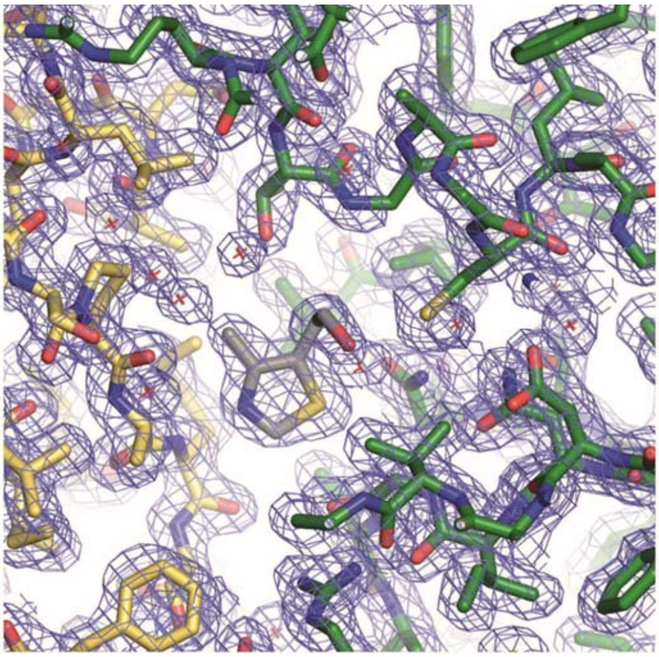

A view of the experimental 2FoFc - electron density of *Sa*ThiM in complex with THZ is given. The map was calculated to 1.90 Å resolution. The active site with the THZ incooperated is revealing the ligand positioning. In this detailed view of one active site region the protein is shown in stick representation with carbon chain atoms in the respective chain color, nitrogen in blue, oxygen in red and sulfur in yellow. THZ is colored grey with the above mentioned element color scheme. The quality of these data ensured the placement of the ligand with high confidence, which was the basis for the onwards substrate analog selection.

Fig. S5: Proposed ATP binding mode to the ThiM active site

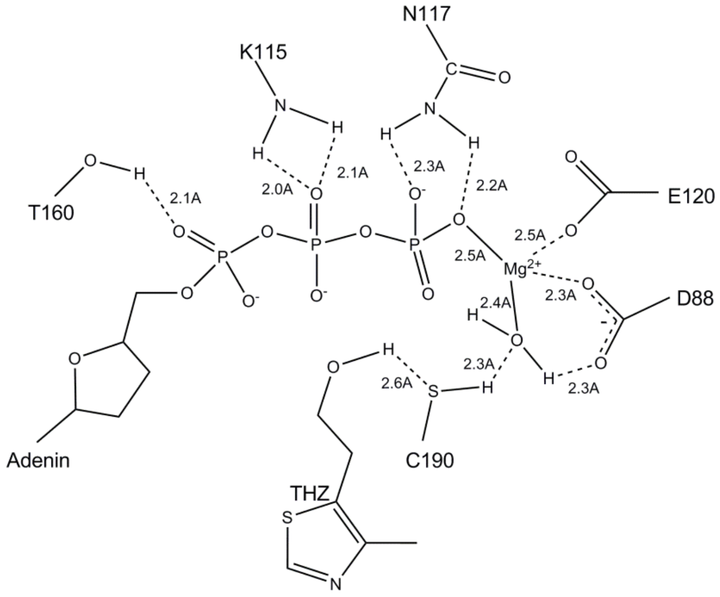

Schematic illustration of the *Sa*ThiM active site with bound ATP and THZ.

Fig. S6: Activity of the compounds

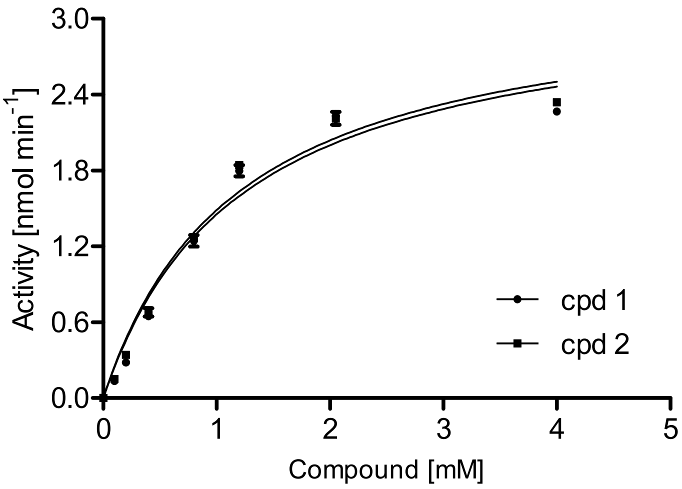

Graphical plot of the Michaelis-Menten kinetics of compound 1 (cpd 1) and compound 2 (cpd 2) illustrating the acceptance of both compounds as substrates. Calculation of the kinetics was performed using GraphPadPrism software (USA).
